# Validation of IMU-based gait event detection during curved walking and turning in older adults and Parkinson’s Disease patients

**DOI:** 10.1186/s12984-021-00828-0

**Published:** 2021-02-06

**Authors:** Robbin Romijnders, Elke Warmerdam, Clint Hansen, Julius Welzel, Gerhard Schmidt, Walter Maetzler

**Affiliations:** 1 Digital Signal Processing and System Theory, Institute of Electrical and Information Engineering, Kiel University, Kaiserstraße 2, 24143 Kiel, Germany; 2 Neurogeriatrics, Department of Neurology, University Hospital Schleswig-Holstein, Arnold-Heller-Straße 3, Haus D, 24105 Kiel, Germany

**Keywords:** Gait, Gyroscope, Older adults, Parkinson, Step detection, Stroke, Turns, Walking, Wearable sensors

## Abstract

**Background:**

Identification of individual gait events is essential for clinical gait analysis, because it can be used for diagnostic purposes or tracking disease progression in neurological diseases such as Parkinson’s disease. Previous research has shown that gait events can be detected from a shank-mounted inertial measurement unit (IMU), however detection performance was often evaluated only from straight-line walking. For use in daily life, the detection performance needs to be evaluated in curved walking and turning as well as in single-task and dual-task conditions.

**Methods:**

Participants (older adults, people with Parkinson’s disease, or people who had suffered from a stroke) performed three different walking trials: (1) straight-line walking, (2) slalom walking, (3) Stroop-and-walk trial. An optical motion capture system was used a reference system. Markers were attached to the heel and toe regions of the shoe, and participants wore IMUs on the lateral sides of both shanks. The angular velocity of the shank IMUs was used to detect instances of initial foot contact (IC) and final foot contact (FC), which were compared to reference values obtained from the marker trajectories.

**Results:**

The detection method showed high recall, precision and F1 scores in different populations for both initial contacts and final contacts during straight-line walking (IC: recall $=$ 100%, precision $=$ 100%, F1 score $=$ 100%; FC: recall $=$ 100%, precision $=$ 100%, F1 score $=$ 100%), slalom walking (IC: recall $=$ 100%, precision $\ge$ 99%, F1 score $=$100%; FC: recall $=$ 100%, precision $\ge$ 99%, F1 score $=$100%), and turning (IC: recall $\ge$ 85%, precision $\ge$ 95%, F1 score $\ge$91%; FC: recall $\ge$ 84%, precision $\ge$ 95%, F1 score $\ge$89%).

**Conclusions:**

Shank-mounted IMUs can be used to detect gait events during straight-line walking, slalom walking and turning. However, more false events were observed during turning and more events were missed during turning. For use in daily life we recommend identifying turning before extracting temporal gait parameters from identified gait events.

## Background

Gait is recognized as a surrogate marker of health, and provides essential clinical insights in neurological disease status [1, 2]. Traditionally, gait has been assessed by visual observation, which suffers from subjectivity and imprecision [3]. To overcome these limitations, multi-camera optical motion capture (OMC) systems can be used, but these systems are relatively expensive and restricted to expertise laboratories [4]. Furthermore, there is increasing evidence that the gait pattern observed in clinical gait assessments does not reflect daily-life gait [5, 6]. Hence, to get a more complete picture of health status, there is an increasing demand for methods that allow for long-term gait monitoring in ambulatory settings. Inertial measurement units (IMUs) provide a promising alternative to assess gait in an objective, unobtrusive and unconstrained manner [4, 7].

The term “gait” refers to “the way of walking” [8, 9] and human gait is commonly segmented into repetitive gait cycles. A normal gait cycle begins and ends with initial contact (IC), the instance when the foot strikes the ground [10]. The time interval between two consecutive ICs of the same foot is referred to as the gait cycle time or stride time. The time interval between two successive ICs of the opposite feet is called the step time. If, additionally, the event of final foot contact (FC) is considered, then all phases in the gait cycle can be described: swing and stance phase, or single and double support phase [1, 10]. Identification of gait events (GEs) and phases is considered essential for clinical gait assessment [8]. GEs can be detected from a single low back-mounted IMU [11–16], however findings suggest that detecting GEs is easier from shank- or foot-mounted IMUs [17–19] where foot-mounted IMUs increase errors, especially in pathological gait patterns [19, 20].

The performance of IMU-based GE detection is, however, often tested only with treadmill walking [12, 14] or from walking trials where only the straight-line segments of walking trajectories were included in the analysis [13, 17, 21]. For more complex walking tasks, such as slalom walking or dual-task walking, one often relies on visually counting of the number of steps, which does not allow to assess the time error of the GE detection and is more prone to errors. Whether IMU-based GE detection is still valid in more complex walking tasks is yet to be shown. Daily-life gait is likely influenced by obstacle negotiation (approximately 30% of daily-life gait is spent along curved trajectories [22, 23]) and dual-/multi-tasking [5].

The aim of this study is therefore to quantify the performance of IC and FC detection in straight-line walking under single-task and dual-task conditions, and to quantify detection performance in curved walking and turning in (healthy) older adults (OA), people diagnosed with Parkinson’s disease (PD), and people who have suffered from a stroke (ST).

## Methods

A step was considered as the interval between consecutive ICs of the ipsi- and contralateral foot [10], and corresponding to forward displacement of the foot together with a forward displacement of the trunk [24]. A stride was the interval between two consecutive ICs of the same foot, and as such it was equivalent to the gait cycle and every stride consisted of two steps [8, 10].

### Study population

Three different groups were distinguished: (1) OAs with no signs of any movement disorders, (2) PD participants in the medication ON state, and (3) ST participants (Table 1). For the OAs the minimum age was 60 years. All participants needed to be able to walk independently with or without walking aids. Exclusion criteria were a high fall risk (i.e. > 2 falls in the last month, as reported by the participant), any impairment that refrained the participant from giving consent to participate in the study, and a score below 20 for the Montreal Cognitive Assessment (MoCA) [25]. All participants gave written informed consent and the study was approved by the ethical committee of the medical faculty at University Hospital Schleswig-Holstein (UKSH), No: D438/18.

**Table 1:** Demographic data of the study participants summarized by group

Group	Participants (female)	Age (years)	Height (m)	Mass (kg)	UPDRS-$\hbox {III}^{1}$	Disease $\hbox {duration}^{2}$ (years)
OA	11 (2)	71±9	1.76±0.07	78.5±13.5	4±3	
PD	14 (5)	64±10	1.78±0.08	91.3±14.7	29±21	9±5
ST	9 (2)	68±10	1.75±0.08	81.3±18.0	6±9	2±4

$^1$ Unified Parkinson’s Disease Rating Scale, part III: Motor Examination, $^2$ for PD: the time since first diagnosis, for ST: the time since stroke

### Study protocol

**Fig. 1:**
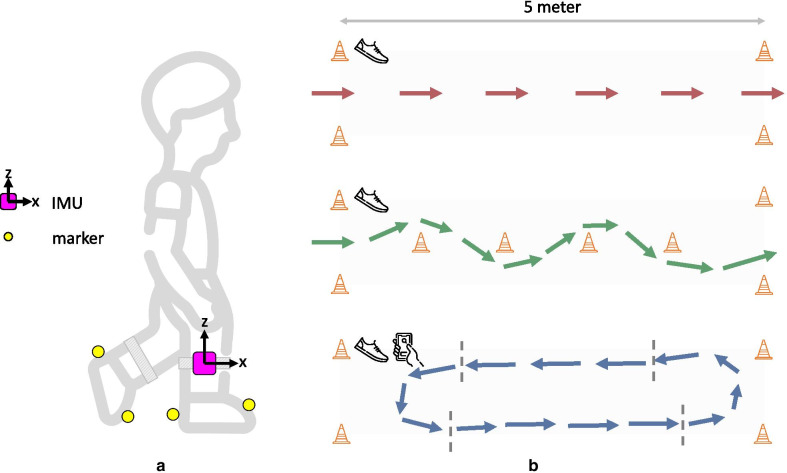
Schematic illustration of the setup. **a** Participants were equipped with inertial measurement units (IMUs) attached to the lateral sides of the shanks and reflective markers that were attached to the heel and toe region of the shoes. **b** Three different walking trials were performed: (top) straight-line walking trial, (middle) slalom walking trial, and (bottom) Stroop dual-task walking trial. Straight-line walking and slalom walking were performed only under single-task conditions (indicated by the shoe icon), whereas the Stroop task was as a cognitive-motor dual-task (indicated by the shoe and mobile phone icons). For the Stroop-and-walk trial, we distinguish between steps during straight-line segments (within the dashed vertical lines) and steps during turns (outside of the dashed vertical lines)

Participants walked a 5-meter distance that was marked at the start and end with two cones, approximately 1 meter apart (Fig. 1). Participants were asked to start walking approximately two steps before the start, and to stop walking approximately two steps after the end. For the analysis of GE detection, we only considered the events that were registered within the 5-meter distance.

The following walking trials were performed:

- straight-line trial, 5 meter, at preferred speed,
- slalom trial, 5 meter with a cone at every 1 meter, at preferred speed,
- Stroop-and-walk trial, walking up and down the 5-meter distance, while performing a numerical Stroop test [26] on a hand-held mobile phone until completion of the Stroop test, at preferred speed. For the numerical Stroop test, two numbers were displayed on the mobile screen that were different in value and different in semantic size. The participant needed to tap the number with the highest value. No further instructions as to prioritize any task were given.

For the Stroop-and-walk trial, participants started within the 5-meter distance, and walked up and down whilst turning on either end of the 5-meter distance (see Fig. 1 for an example trajectory). Turns were annotated manually using the Qualisys Track Manager 2018.1 software (QTM; Qualisys AB, Göteborg, Sweden), and GEs during turns were analyzed separately.

### Optical motion capture system

#### Equipment

Reflective markers (diameter: 19 mm) were attached to the heel and toe of both left and right shoes (Fig. 1). Marker trajectories were recorded by a 12-camera optical motion capture system (Qualysis AB, Götebörg, Sweden) sampling at 200 Hz.

#### Signal processing

GEs were detected from the heel marker trajectories, and provided reference values for the GE timings of ICs and FCs. Event timings were based on specific signal features from the heel marker vertical velocity and acceleration, respectively. Raw marker data were loaded into MATLAB (MATLAB 2018b, The Mathworks, Natwick, USA). The raw marker data were first interpolated to fill any gaps [27] and subsequently low-pass filtered with a 4th order Butterworth filter with a cut-off frequency, $f_{\mathrm {cut}}$, of 5 Hz. The filter was applied to the marker data by using MATLAB’s built-in filtfilt function, such that the filtered signal was not delayed.

The timings of ICs correlated with timings of local maxima in the vertical acceleration [28], whereas the timings of FCs closely correlated with timings of local maxima in the heel marker vertical velocity [29]. Like in [30], GEs were checked manually using QTM.

### Inertial measurement units

#### Equipment

One IMU (Noraxon U.S.A. Inc., Scottsdale, Arizona, USA) was attached to each shank with elastic straps. The sampling frequency was also set to 200 Hz and the OMC and IMUs were synchronized using a trigger at the beginning of each measurement [31].

#### Signal processing

Sensor data were loaded into MATLAB. IMUs were aligned such that the sensitive axes pointed roughly in antero-posterior direction (forward being positive), medio-lateral direction (left being positive), and the vertical direction (up being positive). Angular velocity was high-pass filtered using an IIR filter with $f_{\mathrm {cut}} \approx$ 0.15 Hz to reduce the effect of drift [17], and then low-pass filtered using a 4th order Butterworth filter with $f_{\mathrm {cut}}=$ 10 Hz. Both filters were applied using the filtfilt function.

Detection of GEs using shank-mounted IMUs was based on identifying negative peaks in the medio-lateral angular velocity that was high- and low-pass filtered. These negative peaks closely correlated to timings of mid-swing [17, 18, 32]. Only negative peaks with a value $\le$ 10% of the global minimum angular velocity were considered. Furthermore, if two or more consecutive peaks were detected within a time interval of 300 ms of each other, then only the peak with the lowest value was preserved [21, 33].

Data from the two legs were analyzed independently of one another to facilitate a setup with an IMU on a single side.

### Data analysis

Two GEs, IC and FC, were extracted using the reference system as well as the shank-mounted IMUs. For both events we evaluated the detection performance in terms of correctly identified events (true positives, *TP*), falsely identified events (false positives, *FP*) and missed events (false negatives, *FN*). *TP*s were defined as < 300 ms difference (in terms of magnitude) between an event detected by the IMU-based algorithm and the reference event [12]. From these metrics the recall, precision and F1 score were derived:

1 $$\begin{aligned} \text {Recall} = R&= \frac{TP}{TP+FN} \end{aligned}$$

2 $$\begin{aligned} \text {Precision} = P&= \frac{TP}{TP+FP} \end{aligned}$$

3 $$\begin{aligned} \text {F1 score} = F1&= 2 \frac{P \cdot R}{P + R} \end{aligned}$$

Recall expressed how many of the gait events were detected and precision expressed how many of the detected gait events were true gait events. The F1 score can be considered as a weighted average of the recall and precision. Furthermore, algorithm performance was evaluated by assessing the time error between the reference event (from the marker-based algorithm) and the predicted event [34, 35], defined as:

4 $$\begin{aligned} \epsilon _{\text {IC}}&= t_{\text {IC}} - t'_{\text {IC}} \end{aligned}$$

5 $$\begin{aligned} \epsilon _{\text {FC}}&= t_{\text {FC}} - t'_{\text {FC}} \end{aligned}$$

where $t_{\text {IC}}$ and $t_{\text {FC}}$ denoted the time of the predicted IC and FC from the IMU-based algorithm, and $t'_{\text {IC}}$ and $t'_{\text {FC}}$ denoted the reference time of the IC and FC obtained from the OMC.

The effect of dual-task conditions was investigated by comparing GE detection from the straight-line trial to the GE detection from the straight-line segment of the Stroop-and-walk trial (Fig. 1). The effect of curved walking was investigated by comparing GE detection from the straight-line walking to slalom walking and turns from the Stroop-and-walk trial.

### Statistical analysis

#### Detection of gait events

The algorithm performance in detection of GEs was evaluated by generating contingency tables and comparing the recall, precision and F1 scores.

#### Time agreement

Time agreement was assessed by determining the mean time error, corresponding 95% confidence intervals and the mean absolute error (MAE). Confidence intervals (CIs) were computed as ${\bar{x}} \pm 1.96s$ with ${\bar{x}}$ the mean time error, and *s* the standard deviation of the time errors.

*Comparison of time errors between tasks for each group.* A Wilcoxon signed-rank test [36] was used to compare each subject’s mean values of the absolute errors for the single-task versus the dual-task conditions, and similarly for the straight-line walking, slalom walking, and turns [18]. Differences were considered statistically significant if the p-value was less than 0.05.

*Comparison of time errors between groups for each task.* A Wilcoxon rank sum test was used to compare the subject mean values of the absolute errors from the OA group and those obtained for the PD and ST group [18]. Differences were considered statistically significant if the p-value was less than 0.05.

## Results

### Detection of gait events

#### Effect of dual-tasking

**Table 2:** Validation results of gait event detection for the straight-line segments of the single-task trial and the Stroop-and-walk trial

		**Initial contacts**						**Final contacts**					
	*n*	TP	FN	FP	R	P	F1	TP	FN	FP	R	P	F1
					(%)	(%)	(%)				(%)	(%)	(%)
**Straight-line trial**													
OA	11	83	0	0	100	100	100	83	0	0	100	100	100
PD	14	131	0	0	100	100	100	133	0	0	100	100	100
ST	9	78	0	0	100	100	100	81	0	0	100	100	100
**Stroop-and-walk trial (straight-line segments)**													
OA	11	501	5	0	99	100	100	497	8	1	98	100	99
PD	11	587	1	1	100	100	100	589	2	2	100	100	100
ST	9	457	12	0	97	100	99	451	18	2	96	100	98

IMU-based GE detection showed high recall (IC: $\ge 97\%$, FC: $\ge 96\%$), high precision (IC: $\ge 100\%$, IC: $\ge 100\%$) and high F1 score (IC: $\ge 99\%$, FC: $\ge 98\%$) for the three different groups in both single-task and dual-task conditions for GEs during straight walking (Table 2). All ICs and FCs were detected for the single-task trials. In the straight walking segments from the Stroop-and-walk trials a number of false events (1 IC, 5 FC) and a number of missed events (18 IC, 28 FC) were observed.

#### Effect of curved walking and turns

**Table 3:** Validation results of gait event detection during straight-line walking, curved walking, and turns

		**Initial contacts**						**Final contacts**					
	*n*	TP	FN	FP	R	P	F1	TP	FN	FP	R	P	F1
					(%)	(%)	(%)				(%)	(%)	(%)
**Straight-line trial**													
OA	11	83	0	0	100	100	100	83	0	0	100	100	100
PD	14	131	0	0	100	100	100	133	0	0	100	100	100
ST	9	78	0	0	100	100	100	81	0	0	100	100	100
**Slalom trial**													
OA	11	103	0	0	100	100	100	96	0	0	100	100	100
PD	14	181	0	1	100	99	100	190	0	1	100	99	100
ST	9	124	0	0	100	100	100	126	0	0	100	100	100
**Stroop-and-walk trial (turns)**													
OA	11	297	51	11	85	97	91	296	55	15	84	95	89
PD	11	312	45	18	87	95	91	315	42	14	88	96	92
ST	9	281	41	15	87	95	91	282	39	13	88	96	92

IMU-based GE detection showed high recall (IC: $100\%$, FC: $100\%$), high precision (IC: $\ge 99\%$, FC: $\ge 99\%$) and high F1 score (IC: $100\%$, FC: $100\%$) for the three different groups for the straight-line walking and slalom walking (Table 3). One IC and one FC were falsely detected for a single PD patient in the slalom walking trial, where the patient swung its foot forward and backward without taking a step (that is, there was a swing phase but the patient did not move forward but rather put its foot down on the same spot). For ICs and FCs during turns, for the three groups, recall was lower than for the straigt-line and slalom walking (IC: $\ge 85\%$, FC: $\ge 84\%$), and likewise for the precision (IC: $\ge 95\%$, FC: $\ge 95\%$) and F1 score (IC: $\ge 91\%$, FC: $\ge 89\%$). More events were missed by the IMU-based gait event detection (137 ICs, 136 FCs) and more false events were detected (44 ICs, 42 FCs).

### Time agreement

#### Effect of walking task on gait event detection

**Table 4:** Values for the time errors of the gait events

		**Time error (ms)**					
		Initial contacts			Final contacts		
		Mean$\pm$ $\hbox {sd}^{1}$	MAE	95% CI	Mean$\pm$ $\hbox {sd}^1$	MAE	95% CI
OA	Straight-line	14 ± 36	32	[− 57,85]	− 25±33	31	[− 90,39]
OA	Stroop-and-walk	21 ± 33	32	[− 44,86]	− 21±38	33	[− 96,54]
PD	Straight-line	26 ± 29	33	[− 30,83]	− 25±45	40	[− 113,63]
PD	Stroop-and-walk	11 ± 40	33	[− 67,89]	− 43±49	51	[− 139,52]
ST	Straight-line	17 ± 36	31	[− 54,87]	− 6±33	29	[− 71,59]
ST	Stroop-and-walk	31 ± 43	41	[− 53,116]	− 4±38	32	[− 79,71]

$^1$ sd: standard deviation

**Fig. 2:**
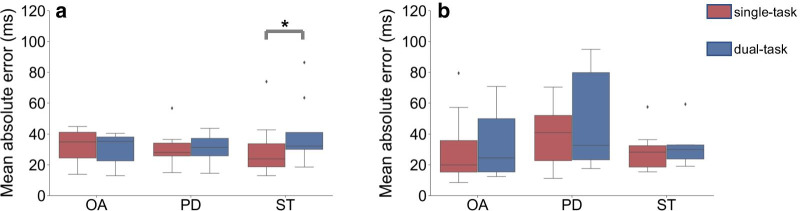
Mean absolute errors. Boxplots showing the mean absolute errors for the **a** initial contacts and **b** final contacts detection for the gait events during straight-line walking under single-task (red) or dual-task (blue) conditions. *: $p<$ 0.05

*Effect of dual-task walking.* Table 4 shows the mean time errors, mean absolute error (MAE) and the 95% CI for the GEs during straight-line walking, either from the straight-line trial (single-task) or the Stroop-and-walk trial (dual-task). A Wilcoxon signed-rank test showed that there are no significant differences between the single-task and dual-task conditions, except for the ST group (Fig. 2). In the ST group we found that the MAE is significantly higher in dual-task conditions compared to single-task conditions (*p*=0.039, *W*=5.0).

**Table 5:** Values for the time errors of the gait events

		**Time error (ms)**					
		Initial contacts			Final contacts		
		Mean ± sd	MAE	95% CI	Mean ± sd	MAE	95% CI
OA	Straight-line	14 ± 36	32	[− 57,85]	− 25 ± 33	31	[− 90,39]
OA	Slalom	18 ± 32	30	[− 45,81]	− 2 ± 31	25	[− 63,59]
OA	Stroop-and-walk (turns)	11 ± 57	40	[− 101,123]	− 33 ± 77	58	[− 183,117]
PD	Straight-line	26 ± 29	33	[− 30,83]	− 25 ± 45	40	[− 113,63]
PD	Slalom	20 ± 33	30	[− 44,83]	− 15 ± 45	35	[− 103,72]
PD	Stroop-and-walk (turns)	− 11 ± 81	56	[− 170,148]	− 45 ± 75	68	[− 192,103]
ST	Straight-line	17 ± 36	31	[− 54,87]	− 6 ± 33	29	[− 71,59]
ST	Slalom	19 ± 36	31	[− 53,90]	− 4 ± 34	28	[− 72,63]
ST	Stroop-and-walk (turns)	12 ± 62	47	[− 110,133]	− 7 ± 77	54	[− 158,144]

**Fig. 3:**
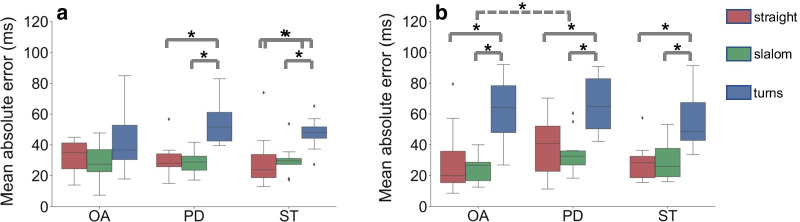
Mean absolute errors. Boxplots showing the mean absolute errors for the **a** initial contacts and **b** final contacts detection for the gait events during straight-line walking (red), slalom walking (green) and during turns (blue). *: $p<$ 0.05

*Effect of curved walking and turns.* Table 5 shows the mean time errors, MAE and 95% CI for GE detection during straight-line walking, slalom walking and during turns. A Wilcoxon signed-rank test showed that for all groups there are no significant differences between the straight-line walking and the slalom walking, for both ICs and FCs. It is also shown that in IC detection significant differences exist for the MAE of the straigt-line walking and turns, and the slalom walking and turn, for both the PD and ST group (Fig. 3). For the FC detection, significant differences were observed for the MAEs between straight-line walking and turns, and slalom walking and turns, for all groups.

#### Effect of group on gait event detection

A Wilcoxon rank-sum test showed that no significant differences were observed between groups for all walking tasks, except for slalom walking, where in FC detection the MAE is significantly larger in the PD group compared to the OA group (*p *= 0.039, *W*= −  2.608).

## Discussion

In this study, shank-mounted IMU-based detection of gait events was tested for different walking tasks and in different mobility-limiting chronic diseases. The detection performance was evaluated for GEs from steps during straight-line walking under both single-task and dual-task conditions. Furthermore, the detection performance was evaluated for GEs from steps during straight-line walking, curved walking and turns. Three different groups of participants were distinguished (OA, PD, ST; Table 1) to evaluate whether the detection performance is affected by presence or history of neurological disease.

The high (i.e., almost perfect) recall, precision and F1 score (Table 2) imply that IMU-based detection of ICs and FCs is feasible in both single-task and dual-task straight-line walking for participants with physiological and different pathological walking patterns. Similarly, the high (i.e., again almost perfect) recall, precision and F1 score (Table 3) imply that IMU-based gait event detection is feasible in curved walking by assessing the detection performance in slalom walking. Again, results hold for participants across different walking conditions. The performance of detecting gait events during turning shows lower recall, precision and F1 score which suggests that shank-mounted IMUs are less feasible to detect GEs during turning. It should be noted that this holds only for how currently the signals were processed. If the vertical acceleration signal would also have been used, like in [37] for lower limb amputees, then GE detection would likely have been less dependent on the (forward) swinging motion of the leg.

Concerning the differences between IMU-based event timings and reference event timings, results were in a range similar to previous studies [30, 35]. There are many possible contributors to the time difference, and possibly a combination of these will be in play. First, and most importantly, two different systems were used and distinct signal characteristics were used to identify the same event. For the reference system, local extrema in the marker velocity and acceleration marked the instances of the event. [28, 29] For the IMUs, local extrema in the angular velocity about the medio-lateral axis marked the instances of gait events. [17, 18, 32] More recent research found that there was no clear feature from the angular velocity signal related to FC, at least when walking on a treadmill. [38] Next, the filtering of the angular velocity signal may contribute to the time error, as with a lower cut-off frequency less signal details were preserved which affects the presence of local extrema. [39] Potentially, there is also a minor contribution to the time error from the hardware-triggered synchronization [31], as mentioned before ( [30, 40, 41]). Most importantly, for all groups and walking tasks, the 95% CI of the estimated event timing encompassed 0 s and therefore the current methods are considered valid for detection of gait events [17, 18, 32]. The 95% CI is largest for the time errors of IC and FC during turns.

It was found that the absolute time error is not significantly different when comparing detection of GEs for single-task conditions to dual-task conditions, except for the detection of ICs for the ST group. This may be explained by the altered gait pattern that can be observed in some post-stroke patients, especially while executing a cognitive-motor dual-task [42, 43]. The mean absolute time error was found significantly higher for IC and FC detection during turns compared to both straight-line and slalom walking for the PD and ST group. Together with the lower recall, precision, F1 score and the larger 95% CI this implies that a shank-mounted IMU is less feasible for detecting GEs during turning. What are the most probable reasons for this observation? In straight-line walking, the leg is swung forward reaching a peak angular velocity at approximately mid-swing [17, 18, 32]. The local maxima right before and after the peak are then correlated to the instances of FC and IC [17, 18, 32]. However, for steps during turning, this swinging motion may not always be observed, depending on which turning strategy is used [44] which may explain the higher number of FN and FP events during turns, compared to straight-line and, especially, slalom walking. The study has in our opinion a particular clinical relevance, considering that increasingly home-derived data will be used for patient management [6] and assessment in clinical trials [45, 46]. IMUs are ideally suited for this performance-based assessment. Our study suggests that the temporal components necessary for the qualitative assessment of gait (IC and FC) can be detected very reliably during straight and slalom walking (e.g. go for a stroll or shopping, commuting to work), but gait phases with rotations of e.g. 180$^{\circ }$ and possibly interrupted forward movement can be detected less reliably. This implies that turns should definitely be included in unsupervised IMU-based gait detection. Of course, this statement only applies to the sensor constellation as used in this study, and not, for example, to data from IMUs positioned at the low back. Although methods derived from low back- or foot-mounted IMUs [12, 13, 20, 47] may be less susceptible to turns, literature suggested that GE detection is easier and more robust from shank-mounted IMUs. [17–19]

Furthermore, to continue improving the current methods, and to be less dependent on the forward swinging motion of the leg, future research may also include vertical acceleration signals [13, 37] or include information from both the time and frequency domain [34, 48].

One of the limitations of this study is the relatively short walking distance. However, the focus of our research was on detecting GEs regardless of the walking distance. Another limitation is that the results are from a supervised assessment in a controlled environment, which is not representative of daily-life conditions [5, 6].

## Conclusion

Shank-mounted IMUs can be used to detect gait events from steps during straight-line and curved walking, under both single-task and dual-task conditions, in different neurological populations. Gait events from steps during turns can be detected but result in more missed events and more false events. In case spatio-temporal parameters are subsequently derived, the higher number of missed and false events will have a negative effect on these parameters. If turns are not automatically identified, the spatio-temporal parameters from ambulatory assessment should be interpreted with care.

## Data Availability

The data that was collected and/or analyzed are not publicly available but are available from the authors on reasonable request.

## Abbreviations

FC: Final contact
FN: False negative
FP: False positive
GE: Gait event
IMU: Inertial measurement unit
IC: Initial contact
MAE: Mean absolute error
MoCA: Montreal Cognitive Assessment
OA: Older adult
OMC: Optical motion capture
PD: Parkinson’s disease
sd: Standard deviation
ST: Stroke
TP: True positive
